# Prevalence of diabetes mellitus amongst hospitalized tuberculosis patients at an Indian tertiary care center: A descriptive analysis

**DOI:** 10.1371/journal.pone.0200838

**Published:** 2018-07-18

**Authors:** Tripti Pande, Sophie Huddart, Wilbin Xavier, Srivathsa Kulavalli, Tanya Chen, Madhukar Pai, Kavitha Saravu

**Affiliations:** 1 McGill International Tuberculosis Centre, Montreal, Canada; 2 Department of Medicine, Kasturba Medical College, Manipal Academy of Higher Education (MAHE), Manipal, Karnataka, India; 3 Faculty of Medicine, McGill University, Montreal, Canada; University of Otago, NEW ZEALAND

## Abstract

**Background:**

India has a high prevalence of tuberculosis (TB) as well as diabetes mellitus (DM). DM is a chronic disease caused by deficiency of insulin production by the pancreas. The risk of TB amongst DM patients is three times higher than those without. The estimated national prevalence of DM is 7.3%. Despite the growing burden of DM, there are limited studies describing the prevalence of TB-DM in India.

**Objective:**

Our study estimated the prevalence of DM amongst adult hospitalized TB patients at Kasturba Hospital, Manipal and determined factors associated with the likelihood of DM-TB co-prevalence.

**Methods:**

We conducted a retrospective cohort study at Kasturba Hospital, Manipal Academy of Higher Education. All hospitalized adult patients diagnosed with pulmonary TB (PTB) and extrapulmonary TB (EPTB) between June 1^st^ 2015 and June 30^th^ 2016 were eligible for inclusion. Pediatric and pregnant TB patients were excluded from our study. Data were extracted from medical charts. Descriptive and multivariate analyses were performed in R. Multivariate analysis adjusted for age, gender, type of TB, history of TB, and nutrition (body mass index (BMI)) status.

**Results:**

A total of 728 patients met the eligibility criteria, 517 (71%) were male, 210 (29%) female, 406 (56%) had PTB and 322 (44%) had EPTB. Amongst those with a nutritional status, 36 (30%) patients were underweight (BMI <18.4 kg/m^2^), 73 (40%) had a normal BMI (18.5kg/m^2^–24.9 kg/m^2^), 15 (8%) were overweight (BMI 25.0 kg/m^2^–29.9 kg/m^2^) and 9 (5%) were obese (BMI >30.0 kg/m^2^). A total of 720 (98.9%) of TB patients had at least one blood sugar test result. The overall prevalence of DM (n = 184) amongst TB patients was 25.3% (95% CI 22.2%, 28.6%). When stratified, it was 35.0% (30.4%, 39.9%) and 13.0% (9.7%, 17.3%) amongst PTB and EPTB patients respectively. TB patients aged 41–60 years had 3.51 times higher odds (aOR 3.51 (2.08, 6.07)) of having DM than patients 40 years or younger. Patients aged 60 years or older had 2.49 times higher odds (aOR 2.49 (1.28, 4.85)) of having DM than younger patients (<40 years). Females had lower odds (aOR 0.80 (0.46, 1.37)) of developing DM than male TB patients and patients with a history of TB had lower odds (aOR 0.73 (0.39, 1.32)) than newly diagnosed TB patients. Additionally, EPTB patients had significantly lower odds (aOR 0.26 (0.15, 0.43)) compared to PTB patients. Underweight patients also had significantly lower odds (aOR 0.25 (0.14, 0.42)) of having DM than normal weight patients.

**Conclusion:**

Our study found a higher prevalence of TB-DM than the national average. TB-DM co-prevalence was significantly associated with age, type of TB and undernutrition. As India’s DM prevalence is expected to rise, TB-DM will become an increasingly important part of the TB epidemic requiring specialized study and care.

## Introduction

Tuberculosis (TB) has been identified as one of the top 10 causes of death globally [1]. According to the World Health Organization (WHO), there were 10.4 million cases of TB in 2017, and 1.8 million deaths [1]. India accounts for 24% of the global TB burden and 29% of deaths [2]. TB mortality can be attributed to many risk factors, including human immunodeficiency virus (HIV/AIDS), diabetes mellitus (DM), smoking and malnutrition [3]. Malnutrition, also classified as under nutrition, contributes to 55% of the TB incidence in India [3].

In 2016, there were an estimated 61 million DM patients in India [4, 5]. The prevalence of DM is increasing worldwide, with the global prevalence expected to double by 2030 [6]. While the national prevalence of DM is estimated to be 7.3% in India, in urban regions it is higher at 11.2% [7]. Previous studies have indicated that the risk of TB amongst DM patients is three times higher than those without DM [8]. With the prevalence of DM projected to increase by 67% by 2035, the co-burden of TB-DM in India may lead to a major public health crisis [9]. The WHO as well as the Revised National Tuberculosis Program (RNTCP) in India, has recommended routine testing of diabetes amongst TB patients, particularly in high TB burden settings [10, 11]. However, a study conducted by Pizzol *et al*., elucidates the need to further explore co-morbidities of TB, such as diabetes, due to insufficient data [12]. Further, there have been limited studies describing the prevalence of TB-DM in India.

## Methods

### Study objectives

Our study aimed to estimate the prevalence of DM amongst adult hospitalized TB patients at Kasturba Hospital, Manipal and determine factors associated with the likelihood of DM-TB co-prevalence. We aimed to: a) estimate the prevalence of DM in hospitalized adult TB patients, and b) assess factors (e.g. age, HIV, BMI) associated with higher prevalence of DM among TB patients.

### Setting, study population, and definitions

We conducted a retrospective cohort study at Kasturba Hospital Manipal, a tertiary teaching hospital affiliated with Manipal Academy of Higher Education located in the south Indian state of Karnataka. Karnataka has an overall TB case notification rate of 98 per 100 000 patients and approximately 40 000 TB patients are treated in private clinics annually [13]. On average, Kasturba Hospital, Manipal registers 950 TB patients per year [14].

All hospitalized adult patients (≥18 years old) diagnosed with pulmonary TB (PTB) or extrapulmonary TB (EPTB) between June 1^st^ 2015 and June 30^th^ 2016 were included in the study. A patient was considered as a PTB case if they had positive bacteriological culture (solid or liquid), sputum-smear test or positive molecular test (e.g. Xpert MTB/RIF) laboratory results. A patient was considered as an EPTB case if they were microbiologically confirmed by either nucleic acid tests, a positive smear for acid fast bacilli (AFB), or by automated liquid culture [15]. A patient with negative microbiological tests for TB, but strong clinical suspicion and other evidence of EPTB, such as compatible imaging findings, histopathological findings, ancillary diagnostic tests or response to anti TB treatment were also considered as an EPTB case [15].

Patients were considered as positive for DM if they had either; a) self-reported history of DM and were on diabetes treatment, b) fasting plasma glucose ≥ 126mg/dL, c) random plasma glucose ≥ 200mg/dL, or d) glycated hemoglobin (HbA1c) ≥ 6.5%, as per the WHO guidelines [16]. These cutoffs were also used to define elevated blood sugar levels in our analysis. Kasturba Hospital, Manipal hosts a laboratory with a National Accreditation Board for Laboratory (NABL) certification. It requires random plasma glucose testing and encourages fasting blood sugar as well as glycated hemoglobin tests to be done amongst all hospitalized TB patients. Glucose tests are conducted using the Hexokinase method and HbA1c is measured using turbidimetric inhibition immunoassay. Patients were considered “current smokers” if they had been smoking 6 months prior to admission or at hospital admission. Patients were considered as “current alcoholics” if they had been consuming excessive alcohol (>14 drinks/week for men, >7drinks/week for women) 6 months prior to admission or at hospital admission [17]. Pediatric patients (<18 years old) and pregnant TB patients were excluded from the study.

### Data collection and analysis

Data were collected through a structured data extraction form, which included sociodemographic information, TB case characteristics, TB case diagnosis, as well as general information on DM, HIV and patient’s nutritional status. Supplemental queries such as lab investigations (hemoglobin count and bacteriological tests) were also reported. The data extraction form was pilot tested and refined amongst 10 preliminary patient records. Data were collected simultaneously by two researchers and inputted into Excel 2016.

Data analysis was performed using Excel 2016 and R. Patients with missing data were excluded from the relevant analyses i.e. a complete case analysis was performed. The primary outcome was prevalence of DM among TB patients. To identify factors associated with TB-DM co-prevalence, a logistic regression model was used to estimate diabetes prevalence odds ratios (ORs) for higher odds of DM-TB co-prevalence, as compared to TB alone. Multivariate analysis, adjusting for age, sex, history of TB, HIV status, type of TB, and nutritional (BMI) status, was conducted to estimate adjusted prevalence ORs (aORs).

The multivariate analysis variables were selected *a priori* as variables that were likely to be associated with prevalence of DM [7, 18–20] and had acceptable missingness rates. Age was categorized as 18–40 years, 41–60 years, 61+ years. BMI was categorized as underweight (<18.4kg/m^2^), normal (18.5 kg/m^2^-24.9 kg/m^2^), overweight (24.9 kg/m^2^-29.9 kg/m^2^), and obese (>30 kg/m^2^).

### Ethics approval

This study was approved by the Kasturba Medical College and Kasturba Hospital, Ethics Committee (ref: IEC 296/2017) in Manipal, India. Since the study used already collected, routinely compiled hospital and lab records, no consent was obtained.

## Results

During the study period, there were 930 total admissions with any type of TB at Kasturba Hospital, Manipal (S1 Data Set). Upon reviewing patient files, 804 were included for initial screening as 126 files had inconclusive or unspecified diagnosis of TB. The following files were excluded for ineligibility; 43 pediatric files, 8 pregnant TB files and 15 microbiologically negative PTB case files. Further, three patients could not be classified as PTB or EPTB, and 7 patients did not have a test performed for DM diagnosis, thus were excluded. This left a total of 728 TB patients in the analysis. A total of 720 (98.9%) patients had at least one blood sugar test result available.

Table 1 below presents characteristics of the study population. Of the included TB population, 517 (71%) patients were male and 210 (29%) female. Upon stratification, 406 (56%) had PTB and 322 (44%) had EPTB. Ninety-eight (13%) PTB patients and 46 (6%) EPTB patients had a history of TB. Amongst the total patient files, 675 (92%) of TB patients were HIV negative. Ten percent of patients were current smokers and 84 (12%) current alcoholics whereas 30 (4%) were former smokers and 26 (4%) were former alcoholics. Co-morbidities were also stratified, where 33 (4%) patients had chronic lung disease and 23 (3%) patients had hypertension. Other co-morbidities such as chronic liver disease, chronic kidney disease, chronic cardiac disease, anemia etc. are presented in Table 1.

**Table 1:** General characteristics of TB population, n = 728.

Indicator	Sub indicator		PTB n = 406	EPTB n = 322	Total n = 728
			Number (%)	Number (%)	Number (%)
**Age**	18–40		140 (19%)	164 (23%)	304 (42%)
	41–60		175 (24%)	107 (15%)	282 (39%)
	61+		85 (12%)	43 (6%)	128 (18%)
	Unknown		6 (1%)	8 (1%)	14 (2%)
**Sex**	Male		307 (42%)	210 (29%)	517 (71%)
	Female		99 (14%)	111 (15%)	210 (29%)
**Past history of TB**	Yes		98 (13%)	46 (6%)	144 (20%)
	No		305 (42%)	270 (38%)	575 (79%)
	Unknown		3 (0%)	6 (1%)	9 (1%)
**Site of ETPB infection**	Pleural Effusion		-	100 (31%)	100 (14%)
	Lymph node		-	56 (17%)	56 (8%)
	Spinal TB		-	43 (13%)	43 (6%)
	Abdominal TB		-	40 (12%)	40 (5%)
	Other		-	72 (22%)	72 (10%)
**HIV status**	HIV positive		5 (1%)	35 (5%)	40 (5%)
	HIV negative		387 (53%)	284 (39%)	671 (92%)
	Unknown		14 (2%)	3 (0%)	17 (2%)
**Patient History**	Smoking	Current	57 (8%)	17 (2%)	74 (10%)
		Former	24 (3%)	6 (1%)	30 (4%)
		Non smoker	118 (16%)	104 (14%)	222 (30%)
		Not documented	207 (28%)	195 (27%)	402 (55%)
	Alcohol	Current	56 (8%)	28 (4%)	84 (12%)
		Former	20 (3%)	6 (1%)	26 (4%)
		Non alcoholic	111 (15%)	90 (12%)	201 (28%)
		Not documented	219 (52%)	198 (27%)	417 (57%)
**Co-morbidities**	Diabetes mellitus		142 (35%)	42 (13%)	184 (25%)
	Chronic lung disease		24 (6%)	9 (3%)	33 (4%)
	Chronic liver disease		6 (1%)	2 (1%)	8 (1%)
	Chronic kidney disease		4 (1%)	4 (1%)	8 (1%)
	Chronic cardiac disease		4 (1%)	4 (1%)	8 (1%)
	Hypertension		16 (4%)	7 (2%)	23 (3%)
	HbsAg+		5 (1%)	4 (1%)	9 (1%)
	Hepatitis C		1 (0%)	1 (0%)	2 (0%)
	Rheumatoid arthritis		2 (0%)	0 (0%)	2 (0%)
	Cancer		5 (1%)	1 (0%)	6 (1%)
	Steroids/drugs		0 (0%)	0 (0%)	4 (1%)
	Other		13 (3%)	12 (4%)	25 (3%)

PTB = pulmonary tuberculosis, EPTB = extra-pulmonary tuberculosis, n = number, HIV = human immunodeficiency virus, TB = tuberculosis, BMI = body mass index, SD = standard deviation, ESR = erythrocyte sedimentation rate

Patient nutritional status identified 218 (30%) patients as underweight and 231 (32%) with a normal BMI (Table 2). Serum albumin levels were abnormal (<3.4 g/dL) for 175 (24%) and normal (3.5–5.5 g/dL) for 151 (21%) of PTB patients. Whereas 75 (10%) of EPTB patients had abnormal serum albumin levels and 145 (20%) had normal levels. Hemoglobin measurements showed 69% of men and 70% of women with a mild to moderate anemia (7–12.9 g/dL). Further, 2% of men and women had severe anemia (<7g/dL).

**Table 2:** Nutritional status of TB patients.

Indicator	Sub indicator	PTB n = 406	EPTB n = 322	Total n = 728
		Number (%)	Number (%)	Number (%)
**Under weight (BMI <18.4 kg/m^2^)**		159 (22%)	59 (8%)	218 (30%)
**Normal (BMI 18.5 kg/m^2^–24.9 kg/m^2^)**		101 (14%)	130 (18%)	231 (32%)
**Overweight (BMI 25 kg/m^2^–29.9 kg/m^2^)**		17 (2%)	23 (3%)	40 (5%)
**Obese (BMI >30 kg/m^2^)**		7 (1%)	12 (2%)	19 (3%)
**Unknown**		122 (17%)	98 (13%)	220 (30%)
**Serum albumin g/dL**	Abnormal (<3.4)	175 (24%)	75 (10%)	250 (34%)
	Normal (3.5–5.5)	151 (21%)	145 (20%)	296 (41%)
	Unknown	80 (11%)	102 (14%)	182 (25%)
Hemoglobin g/dL*	**Male (n = 517)**			
	Severe anemia (<7)	7 (1%)	2 (0%)	9 (2%)
	Mild to moderate anemia (7–12.9)	226 (44%)	129 (25%)	355 (69%)
	Normal (>13)	60 (12%)	71 (14%)	131 (25%)
	Unknown	14 (3%)	8 (2%)	22 (4%)
	**Female (n = 211)**			
	Severe anemia (<7)	1 (0%)	4 (2%)	5 (2%)
	Mild to moderate anemia (7–11.9)	71 (34%)	75 (36%)	146 (70%)
	Normal (>12)	22 (10%)	27 (13%)	49 (23%)
	Unknown	5 (2%)	5 (2%)	10 (5%)

PTB = pulmonary tuberculosis, EPTB = extra-pulmonary tuberculosis, n = number, HIV = human immunodeficiency virus, TB = tuberculosis, BMI = body mass index, SD = standard deviation, ESR = erythrocyte sedimentation rate

*Denominators for hemoglobin percentages are the respective gender totals.

The prevalence of DM (n = 184) amongst 728 TB patients was 25.3% (95% CI 22.2%, 28.6%). When stratifying between PTB (n = 142) and EPTB (n = 42), the prevalence of DM was 35.0% (30.4%, 39.9%) and 13.0% (9.7%, 17.3%) respectively. Ninety-five percent of TB-DM patients were HIV negative and 12% were current smokers. Forty percent of the patients with TB-DM had normal BMI’s (Table 3). Table 4 provides data on blood sugar levels among TB-DM patients. There were 184 (25.3%) DM patients where glycated hemoglobin testing had been performed in 152 (82%), random blood sugar in 121 (66%), fasting blood sugar in 120 (71%) and post prandial blood sugar in 100 (54%). Amongst these patients, elevated measures were 90.8% (84.7%, 94.7%), 50.4% (41.2%, 59.6%), 66.2% (57.3%, 74.1%), 60.0% (49.7%, 69.5%) for hemoglobin, random blood sugar, fasting blood sugar and post prandial blood sugar tests respectively. The median values [interquartile range (IQR)] for hemoglobin, random blood sugar, fasting blood sugar and post prandial blood sugar were 9.2 [3.65], 210 [184.00], 149 [118.25], and 227 [160.75], respectively.

**Table 3:** General characteristics of TB-DM population (N = 184).

Indicator	Sub-indicator	PTB n = 142	EPTB n = 42	TOTAL n = 184
		Number (%)	Number (%)	Number (%)
**Age**	18–40 years	28 (15%)	7 (4%)	35 (19%)
	41–60 years	75 (41%)	23 (12%)	98 (53%)
	61+ years	36 (20%)	11 (6%)	47 (26%)
	Unknown	3 (2%)	1 (1%)	4 (2%)
**Gender**	Male	114 (62%)	28 (15%)	142 (77%)
	Female	28 (15%)	14 (8%)	42 (23%)
	Unknown	0 (0%)	0 (0%)	0 (0%)
**HIV status**	Positive	0 (0%)	2 (1%)	2 (1%)
	Negative	136 (74%)	39 (21%)	175 (95%)
	Unknown	6 (3%)	1 (1%)	7 (4%)
**Past history of TB**	Present	28 (15%)	9 (5%)	37 (20%)
	Absent	114 (62%)	32 (17%)	146 (79%)
	Unknown	0 (0%)	1 (1%)	1 (1%)
**Smoking**	Current	21 (11%)	2 (1%)	23 (12%)
	Former	8 (4%)	0 (0%)	8 (4%)
	No	47 (26%)	11 (6%)	58 (32%)
	Unknown	66 (36%)	29 (16%)	95 (52%)
**Alcohol**	Current	20 (11%)	4 (2%)	24 (13%)
	Former	8 (4%)	0 (0%)	8 (4%)
	No	44 (24%)	9 (5%)	53 (29%)
	Unknown	70 (38%)	29 (16%)	99 (54%)
**Nutritional status**	Under weight (BMI <18.4)	32 (17%)	4 (2%)	36 (20%)
	Normal (BMI 18.5–24.9)	50 (27%)	23 (12%)	73 (40%)
	Over weight (BMI 25–29.9)	13 (7%)	2 (1%)	15 (8%)
	Obese (BMI >30)	4 (2%)	5 (3%)	9 (5%)
	Unknown	43 (23%)	8 (4%)	51 (28%)
**Glycated hemoglobin (HbA1c)**	4.5% -6.4%	7 (4%)	6 (3%)	13 (7%)
	6.5%– 17.0%	111 (60%)	28 (15%)	139 (76%)
	Not documented	24 (13%)	8 (4%)	31 (17%)

PTB = pulmonary tuberculosis, EPTB = extra-pulmonary tuberculosis, n = number, HIV = human immunodeficiency virus, TB = tuberculosis, BMI = body mass index, SD = standard deviation

**Table 4:** Blood sugar levels amongst TB-DM patient population, n = 184.

Indicator	Number (n)	PTB Median(IQR)	EPTB Median (IQR)	Total Median (IQR)	Proportion of patients with elevated measure (CI)*
**Glycated hemoglobin (HbA1c), %**	152	9.5 (3.375)	7.1 (2.25)	9.2 (3.65)	90.8% (84.7%, 94.7%)
**Random blood sugar, g/dL**	121	222.5 (198)	138 (126)	210 (184)	50.4% (41.2%, 59.6%)
**Fasting blood sugar, g/dL**	130	167 (130.75)	117 (53)	149 (118.25)	66.2% (57.3%, 74.1%)
**Post prandial blood sugar, g/dL**	100	247.5 (173.5)	194 (92.5)	227.5 (166.75)	60.0% (49.7%, 69.5%)

PTB = pulmonary tuberculosis, EPTB = extra-pulmonary tuberculosis, n = number, IQR = interquartile range, CI = 95% confidence interval

*Elevated levels were determined based on the following cutoffs; glycated hemoglobin (HbA1c) >6.5%, random blood sugar >200g/dL, fasting blood sugar >126 g/dL, and post prandial blood sugar >200g/dL.

Table 5 provides crude and adjusted ORs of having TB DM versus having TB without DM across key covariates. When conducting the multivariate analysis, only 484 files were included as they contained complete information for all model variables. The average age of these patients was 44.1 years and 71.5% were male. The average age of the 244 patients excluded due to variable missingness from the model was 45.7 years and 70.1% were male. Older patients had significantly higher odds of having TB-DM; patients older than 60 years had an aOR of 2.49 (1.28, 4.85) compared to 18–40-year old patients. Women were not significantly less likely to have TB-DM than men (aOR 0.80, CI: 0.46, 1.37). EPTB patients had significantly lower odds of TB-DM, with an adjusted OR of 0.26 (0.15, 0.43), compared to PTB patients. Underweight patients had significantly lower odds of TB-DM (aOR 0.25, CI:0.14, 0.42) compared to normal BMI patients but the odds of TB-DM were not significantly higher for overweight or obese patients. To note, most of the excluded patients (90.2%) were excluded for a missing BMI value.

**Table 5:** Comparison of TB DM patients to TB without DM.

Indicator	Sub-indicator	TB with DM n = 184	TB without DM n = 544	Crude Prevalence OR (95% CI)	Adj. Prevalence OR (95% CI)* n = 484
		Number (%)	Number (%)		
**Age**	18–40 years	35 (5%)	269 (37%)	ref	ref
	41–60 years	98 (13%)	184 (25%)	4.09 (2.69, 6.35)	3.51 (2.08, 6.07)
	61+ years	47 (6%)	81 (11%)	4.46 (2.71, 7.42)	2.49 (1.28, 4.85)
	Unknown	4 (1%)	10 (1%)		
**Gender**	Male	142 (20%)	375 (52%)	ref	ref
	Female	42 (6%)	168 (23%)	0.66 (0.44, 0.97)	0.80 (0.46, 1.37)
	Unknown	0 (0%)	1 (0%)		
**History of TB**	No	146 (20%)	429 (59%)	ref	ref
	Yes	37 (5%)	107 (15%)	1.02 (0.66, 1.53)	0.73 (0.39, 1.32)
	Unknown	1 (0%)	8 (1%)		
**Case type**	PTB	142 (20%)	264 (36%)	ref	ref
	EPTB	43 (6%)	280 (38%)	0.28 (0.18, 0.41)	0.26 (0.15, 0.43)
**HIV infection**	Positive	2 (0%)	38 (5%)	0.15 (0.02, 0.49)	0.29 (0.04, 1.09)
	Negative	175 (24%)	496 (68%)	ref	ref
	Unknown	7 (1%)	10 (1%)		
**Nutritional status**	Under weight (BMI <18.4)	36 (5%)	182 (25%)	0.43 (0.27, 0.67)	0.25 (0.14, 0.42)
	Normal (BMI 18.5–24.9)	73 (10%)	158 (22%)	ref	ref
	Overweight (BMI 25–29.9)	15 (2%)	25 (3%)	1.30 (0.63, 2.58)	1.68 (0.74, 3.77)
	Obese (BMI >30)	9 (1%)	10 (1%)	1.95 (0.74, 5.03)	2.28 (0.74, 7.07)
	Unknown	51 (7%)	169 (23%)		

TB = tuberculosis, DM = diabetes mellitus, OR = odds ratio, Adj = adjusted, 95% CI = 95% confidence interval, PTB = pulmonary tuberculosis, EPTB = extra-pulmonary tuberculosis, BMI = body mass index, ref = reference variable used for odds ratio

*484 patients were used for the multivariate analysis due to missingness

## Discussion

Currently, there are an estimated 61 million people diagnosed with DM in India where the estimated prevalence ranges from 5.2% to 12.4% [4, 21, 22]. Our study found a prevalence of DM amongst TB patients of 25.3% (22.2%, 28.6%), which is higher than that of the general population of India. This could be due to the fact that all patients were hospitalized in an urban, tertiary hospital, representing a highly selected group. As shown by Li *et al*. hospitalized TB patients have a significantly higher prevalence of DM compared to patients receiving outpatient care [23]. Previous studies have shown varying results for example three studies in three different Indian states showed prevalence estimates of 19.6% (Kerala), 25% (Tamil Nadu) and 29% (Puducherry) respectively among TB patients [6, 24, 25]. Two of those studies were conducted in hospital settings [6, 24], and one was conducted in an urban community setting [25]. Other studies in China and Saudi Arabia showed an overall prevalence of 12% and 27% respectively amongst TB patients [23, 26]. The study in China further stratified the prevalence amongst rural/urban settings and hospital/TB clinics. There was a higher prevalence of DM amongst TB patients in urban settings (14.0%) and hospitals (13.5%) than rural (10.6%) and TB clinics (8.5%) [23]. To provide a more generalizable analysis, our study would have benefitted from including out-patients as well as hospitalized TB patients. Although most studies have shown a similar prevalence of DM amongst TB patients, each study may have a different method of diagnosing DM. The WHO collaborative framework for care and control of diabetes, which states that screening and diagnostic tests for DM should be adapted to local settings/available resources, may contribute to the heterogeneity of DM diagnostic criteria [27]. Nevertheless, a review conducted by Pizzol *et al*. emphasizes the need to conduct diabetes screening amongst all TB patients, and optimize diabetes management during TB treatment to help increase a patient’s overall health [28].

Our study found that older TB patients (41–60 years) had significantly higher odds of having DM compared to younger patients (<40 years). This is consistent with a nationwide cross-sectional study conducted by Geldsetzer *et al*. which found that the prevalence of DM is highest in middle to older age patients across India [29]. Further, a systematic review conducted by Workneh *et al*. identified 22 studies which indicated that the likelihood of TM-DM comorbidity increases with age [30]. While our study does not find a monotonic increase in the aORs of DM as age increases, the large and overlapping confidence intervals for these estimates suggest that our study may not have sufficient power to precisely estimate the age-DM relationship in this population.

Although our study did not show significant results for gender analysis, male patients had a higher prevalence of TB-DM (77%) than female patients (23%). Similar prevalence estimates have been shown in three other studies, where males had a higher prevalence of TB-DM than females [31–33]. These studies were conducted in India and Pakistan. According to a study conducted by Lawson *et al*. young adult males are generally reported to have a higher risk of TB-DM than females and older individuals [34–36]. All aforementioned studies have been conducted in patriarchal societies thus the higher prevalence of TB-DM in males could be due to increased access to healthcare, as well as higher consumption of tobacco, alcohol and more smoking habits, among males [37].

Underweight patients had significantly lower odds of having DM compared to healthy weight patients whereas overweight and obese patients were not significantly more likely to have DM. There were very few patients who were overweight and obese in our dataset, likely due to TB-related wasting. These sparse strata reduced the power of the comparison of overweight and obese patients to normal BMI patients. Previous studies have shown a significantly higher BMI in patients with TB-DM than those without DM [6, 38]. In contrast, Kumpatla *et al*. reported a lower mean BMI in the TB-DM group as compared to TB without DM [24]. A few studies have reported results similar to our study, where there is no significant relation between BMI and TB-DM [25, 39]. Although previous studies have shown mixed results, our results must be interpreted with caution as our study population was hospitalized TB patients, who are on average sicker than non-hospitalized TB patients. Further, the body weight was recorded at the time of TB diagnosis thus diabetic patients recorded as normal BMI, may have been overweight patients who have lost weight and slipped into this category. The latter further emphasizes the need for a longitudinal prospective study.

When conducting a stratified analysis between EPTB and PTB, our study revealed that EPTB patients had significantly lower odds of having TB-DM. Previous studies conducted in India [37, 40–41], Brazil [42] and the United States of America [43] have shown similar results [42, 43]. Additionally, a study conducted in China demonstrated a higher incidence of DM amongst patients with sputum positive PTB [44]. This is also represented in our results where the prevalence of DM was lower among EPTB patients (13.0%) than PTB (35.0%). A plausible mechanism explaining this association has not yet been sufficiently described.

Poor glycemic control has been associated with an increased risk of TB-DM [45, 46]. Hemoglobin (HbA1c) levels allow for a longer term view whereas glucose levels provide insight into a shorter window. In our study population, 90.8% (84.7%, 94.7%) of the TB-DM patients who had received a HbA1c test had poor glycemic control. A study conducted in China demonstrated that patients with DM presenting with poor glycemic control had a higher hazards ratio for developing TB than patients without DM [46]. Further, a study conducted by Lee *et al*. identified DM patients with poor glycemic control to have double the risk of developing TB than non-diabetics [45]. However, these results must be interpreted with caution as not all patients received a HbA1c test, despite the KMC hospital guidelines indicating the latter.

Our TB-DM study population did not have a high HIV burden; only 1% tested as HIV positive. Upon adjusting for all other variables, a positive HIV status was not significantly related to the odds of having TB-DM. Some researchers argue that the lack of association between TB-DM and HIV may be due to study design [34, 47]. A study conducted by Isa *et al*. showed patients with HIV initiating ART to have a higher risk of developing TB-DM [47]. Given the low numbers of HIV positive patients in our study, we did not have sufficient power to detect an association between HIV and TB-DM.

Our study’s main strength is its large cohort size of 728 patient files diagnosed with TB, allowing for a precise estimate of DM prevalence in this patient population. Despite the large sample size, our study was limited by its retrospective nature, and missing data on covariates. For example, not all patients received the same tests for DM thus we were unable to do a stratified analysis based on the method of DM diagnosis. Also, we were not able to stratify between previously diagnosed DM and new DM cases, due to insufficient data. Further, other variables were not available like data regarding oral hypoglycemic agents (OHA) intake or insulin use, and the family history of TB patients. A systematic review emphasized higher TB-DM comorbidity amongst patients with a family history of TB [30]. In future studies, we would encourage collecting patient and family TB history as key variables and would encourage the use of a standard set of DM diagnostics to administer to TB patients. Further, we could not stratify based on the different forms of diabetes, such as; prediabetes, stress diabetes etc.

Our study may also be subject to selection bias in two ways. First, our study population is hospitalized TB patients at a tertiary care center. These patients were probably sicker than those receiving outpatient TB care and may not represent the general TB patient population. The India tuberculosis and diabetes mellitus study group indicates that higher rates of DM were found among patients with a known history of TB, in South Indian hospitals [48]. A study conducted by Viswanathan et al. presented higher prevalence of DM in the study cohort (25.3%, TB units in South India) as compared to the general population (10.4%) [37, 49]. Studies conducted in hospital settings may result in higher DM diagnosis, as patients are subjected to many screening tests, including DM, once admitted [48]. Second, some variables had high rates of missingness. Approximately 30% of patients lacked BMI measurements and patients without diabetes may have been less likely to have this value recorded. Similarly, patients least likely to have HIV may have been more likely to have a missing HIV status. Additionally, not all patients were directly asked about alcohol usage and smoking. The multivariate analysis was limited to 484 patients with a complete set of covariates.

## Conclusion

Overall, our study has demonstrated a high prevalence of TB-DM co-burden of disease in our tertiary hospital settings. The odds of TB-DM were significantly associated with age, EPTB status and undernutrition (BMI <18.4 kg/m^2^) after adjusting for demographic variables. Our study results are applicable to hospitalized patients. Further, a retrospective cohort study design led to higher rates of missingness amongst many key variables. We therefore recommend that future studies focus on prospectively assessing nutritional status, measuring weight gain, and ensuring proper DM diagnosis for all included patients. We would also encourage a longitudinal study observing both out-patients and in-patients. This would permit for a more comprehensive analysis of TB-DM and ensure targeted solutions.

## Supporting information

S1 Data Set Raw data set of adult TB patients at Kasturba Hospital, Manipal. (XLSX)
